# Modulation of HIV-1 macrophage-tropism among R5 envelopes occurs before detection of neutralizing antibodies

**DOI:** 10.1186/1742-4690-7-48

**Published:** 2010-05-27

**Authors:** Kathryn H Richards, Marlén MI Aasa-Chapman, Áine McKnight, Paul R Clapham

**Affiliations:** 1 Program in Molecular Medicine and Department of Molecular Genetics and Microbiology, University of Massachusetts Medical School, Biotech 2, 373 Plantation Street, Worcester, MA 01605, USA; 2 MRC/UCL Centre for Medical Molecular Virology, Division of Infection and Immunity, University College London, 46 Cleveland Street, London W1T 4JF, UK; 3 Centre for Infectious Disease, Institute of Cell and Molecular Science, Barts and The London, Queen Mary's School of Medicine and Dentistry, The Blizard Building, 4 Newark Street, Whitechapel, London E1 2AT, UK; 4 Institute of Molecular and Cellular Biology, Faculty of Biological Sciences and Astbury Centre for Structural Molecular Biology, University of Leeds, Leeds LS2 9JT, UK

## Abstract

HIV-1 R5 viruses vary widely in their capacity to infect primary macrophages. R5 macrophage-tropism is associated with an increased envelope:CD4 affinity that partly results from an increased exposure of CD4 contact residues on gp120 and allows the use of low levels of CD4 for infection. The selective pressures *in vivo *that modulate R5 macrophage-tropism are not understood. It is possible that different R5 variants adapt for replication in either T-cells (high CD4) or in macrophages (low CD4). However, other selective pressures *in vivo *(e.g. neutralizing antibodies) may also impact R5 tropism. Here, we measured macrophage infectivity conferred by gp120 sequences amplified sequentially from subjects in London followed from the acute stage of infection. We report wide variation in the capacity of these envelopes to confer macrophage infection in the complete absence of both autologous and heterologous neutralizing antibodies. Our data show that the variation in macrophage tropism observed at early times cannot have been influenced by neutralizing antibodies.

## Findings

HIV-1 R5 viruses that use CCR5 (R5) as a coreceptor are preferentially transmitted. Although such viruses are often termed macrophage-tropic or M-tropic [1], we and others have described a wide variation in their capacity to infect primary macrophages [2-7]. In particular, we showed that R5 envelopes amplified from brain tissue of subjects with neurological complications were frequently highly macrophage-tropic (mac-tropic), while many of those from immune tissue, blood, and semen infected macrophages inefficiently [3]. The capacity of R5 envelopes to confer infection of macrophages correlated with their sensitivity to inhibitors that blocked envelope: CD4 interactions, but not with those targeting envelope:CCR5 interactions or gp41 conformational changes [8]. These observations indicated that R5 mac-tropism was modulated by changes in the affinity of envelope for CD4. R5 mac-tropism also correlated with the capacity of envelopes to infect cells via low levels of CD4 [2,3,9,10] consistent with infection of macrophages that express substantially lower amounts of CD4 compared to T-cells [11-13]. In agreement with these observations, we and others have identified gp120 determinants within or proximal to the CD4 binding site (CD4bs) that modulate R5 mac-tropism [11-16].

The selective pressures *in vivo *that drive changes in the mac-tropism of R5 envelopes are poorly understood. It is possible that the different R5 mac-tropic phenotypes result from adaptation for replication in T-cells (high CD4) or in macrophages (low CD4). However, R5 mac-tropism forms a spectrum rather than two separate phenotypes. Thus, other selective pressures *in vivo *need to be considered including neutralizing antibodies (nabs). We previously reported a trend where mac-tropic R5 envelopes were more sensitive to the CD4bs monoclonal antibody, b12, while Dunfee *et al*. reported a significant correlation for envelopes derived from brain and lymph node tissue [17]. Thus, the presence of CD4bs antibodies *in vivo *may select for variants where the CD4bs is protected from neutralization. This possibility was supported by our identification of determinants on the flanks of the CD4 binding loop that modulate mac-tropism and affect b12 sensitivity [14,18]. Such determinants presumably affect the exposure of proximal CD4 contact residues on the CD4 binding loop, which is likely the first contact for CD4 [19]. The protection of these CD4 contact residues from antibodies may compromise the affinity of envelope for CD4 and in turn restrict tropism to cells expressing higher levels of CD4 (e.g. T-cells). In support of this hypothesis, Ryzhova *et al*. reported that the presence of nabs in the CSF correlated with the absence of M-tropic SIVs in rhesus macaques [20]. The predominance of highly mac-tropic envelopes in brain tissue could also reflect adaptation in an immuno-privileged site where antibodies are excluded by the blood brain barrier and usually reach only low concentrations [21,22]. However, brain macrophages and microglia are the predominant targets for HIV-1 in the brain, and the presence of highly mac-tropic variants there may simply reflect an adaptation for infection of these low CD4 cell types.

Here, we have investigated mac-tropism of gp120 sequences amplified sequentially from subjects in London followed from the acute stage of infection. We report wide variation in the capacity of 'early' envelopes to confer macrophage infection in the complete absence of nabs.

We investigated 36 gp120s amplified from three subjects (Table 1) sampled from 12 days to over 7 years after the onset of acute phase symptoms. At early times, gp120s were amplified by nested PCR from proviral DNA in PBMCs, as this was a sensitive approach. At later times, gp120s were amplified from viral RNA in plasma to avoid accumulated archival proviruses and when PCR sensitivity was not an issue. The gp120 sequences were cloned into pHXB2 MCSΔenv via unique Bst EII and Mlu I restriction sites [23]. Replication competent virus was harvested from 293T cells 48 hours after transfection. Infectivity was then estimated by titration on HeLa TZM-bl cells and at least two batches of primary macrophages. In addition, sequential serum samples from the same subjects were tested for neutralization of env+ pseudovirions carrying autologous or heterologous envelopes.

**Table 1:** Subject Details: Viral Load, CD4 counts and envelope PCR

**MM1**	Day^*a*^	**21**	**28**	**48**	**84**	**195**	**494**	**833**	**1231**	**1879**	**2702**
	VL^*b*^	81,000	34,400	14,900	5,400	60,000	63,400	169,300	32,100	111,600	22,200
	CD4^*c*^	880	nd^*d*^	nd	1000	nd	660	830	740	690	530
	env PCR^*e*^		DNA		DNA			RNA			RNA
											
**MM4**	Day	**17**	**52**	**108**	**206**	**297**	**493**	**574**	**844**	**1058**	**1191**
	VL	160,000	9,900	42,300	30,200	24,000	19,900	34,500	137,200	nd	233,400
	CD4	nd	990	590	750	610	690	610	650	nd	490
	env PCR	DNA					RNA		RNA		
											
**MM8**	Day	**12**	**32**	**49**	**81**	**200**	**333**	**608**	**810**	**957**	
	VL	5,927,000	nd	454,100	41,900	59,000	44,500	41,800	105,200	154,800	
	CD4	290	nd	610	350	410	420	260	240	90	
	env PCR	DNA	DNA					RNA		RNA	

*a*. Days counted from onset of symptoms characteristic of primary HIV infection (fever, myalgias, lethargy, a sore throat, headaches, anorexia, diarrhea).

*b*. VL, plasma viral load (RNA copies/ml) determined using Chiron 3.0 (Emeryville, Cal. USA).

*c*. CD4, CD4 cell numbers (cells/μl).

*d*. nd, not determined.

*e*. DNA or RNA source of envelope PCR.

**Figure 1:**
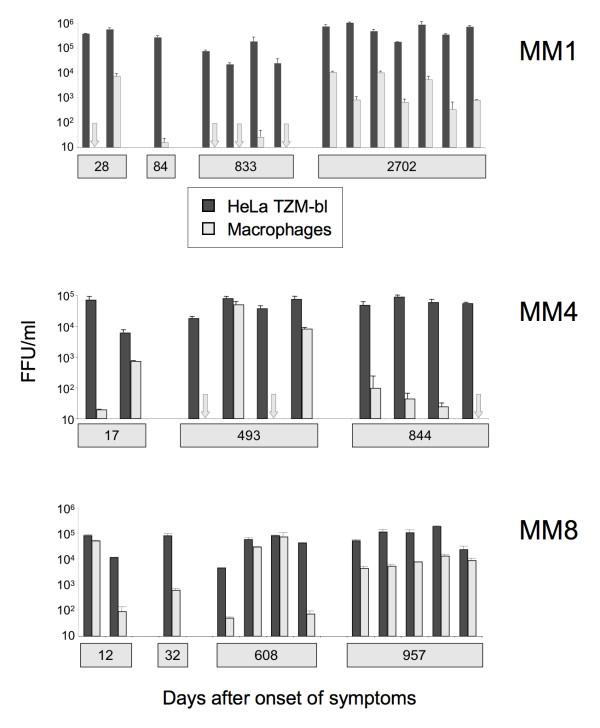
**Macrophage infectivity conferred by HIV-1 gp120 sequences amplified from the acute phase of replication and at various times post-seroconversion**. Replication competent HIV-1 clones carrying gp120 sequences amplified sequentially from subjects MM1, MM4 and MM8 in London were tested for infection of HeLa TZM-bl cells and primary macrophages.

All 36 gp120 sequences investigated conferred efficient infection of HeLa TZM-bl cells that express high levels of CD4 [24] (Figure 1). All envelopes conferred an R5 phenotype except for those from MM8 day 957 which were R5X4 (not shown). We observed extensive variation in macrophage infectivity among the envelopes. For MM1, two envelopes from 28 days after the onset of acute phase symptoms conferred highly divergent levels of macrophage infectivity (Figure 1). Envelopes amplified from 84 and 833 days conferred only very inefficient infection of macrophages, while more substantial levels macrophage infection were observed with envelopes amplified after several years (day 2702) of infection. Similar variation in macrophage infectivity was observed with envelopes amplified from subjects MM4 and MM8. Of note, envelopes that varied dramatically in their capacities to infect macrophages were amplified from the same time point at days 17 and 493 for MM4, days 12 and 608 for MM8 and (as already stated) day 28 for MM1. In contrast envelopes from later time points (day 2702 for MM1, day 844 for MM4, and day 957 for MM8) conferred more uniform levels of macrophage infectivity, with MM8 envelopes imparting higher levels of macrophage infection compared to those from MM4.

Envelope residues that modulate macrophage tropism have been reported to reside within or proximal to CD4 contact residues [14,15]. We examined closely related pairs of gp120 sequences that differed in macrophage infectivity for amino acid differences within or close to the CD4bs that might be responsible for the shift in tropism. Such differences were readily apparent even though each pair of gp120 sequences had relatively few amino acid changes (Table 2). Differences in CD4 contact residues were identified for MM4 and MM8 envelopes and in the V3 loop for MM1 (Table 2). Confirmation of whether these residues are responsible for the shifts in mac-tropism observed will, however, require mutagenesis studies.

**Table 2:** Envelope residues potentially involved in modulating macrophage-tropism

Subject	Days post onset of symptoms	Env clone	Mac infn	Potential amino acid tropism determinants		
				No. amino acid changes in gp120	Env region	Amino acid changes
MM1	28	1.2.1	-	5	V3 loop	NNSRKGIHIGPGRAFY
		1.2.3	+			--T-----------L-

MM4	17	4.1.33	-	5	β24-α5	GGD^1^
		4.1.34	+			--N
	
	493	4.10.1	-	3	β24-α5	GGDMG^1^
		4.10.3	+			----R
MM8	608	8.8.1	-	11	β20-β21	NRWQEA^1^
		8.8.3	+			-----V

1. Amino acids that are CD4 contact residues are underlined on upper sequences [26].

**Figure 2:**
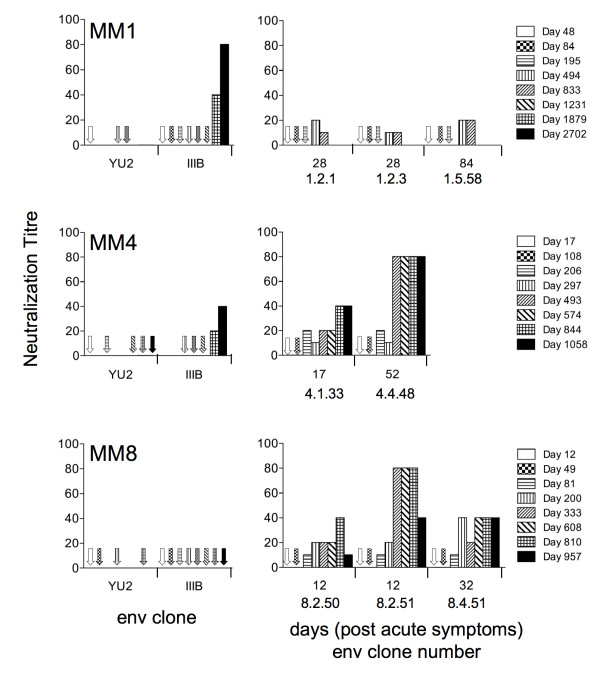
**Detection of neutralizing antibodies present in the plasma of subjects who yielded gp120 sequences studied here**. Serially collected plasma samples (heat inactiviated) were tested for neutralization of YU2 and HTLV-IIIB, a sensitive T-cell line adapted HIV-1 strain (left panels). The same plasma samples were tested for neutralization of viral clones carrying autologous gp120s from the early stages of infection (right panels). Missing arrows or bars means not tested.

Heat inactivated plasma taken during and following the acute stage of infection were tested for the presence of autologous and heterologous nabs. We first investigated whether we could detect nabs that neutralized heterologous viruses (Figure 2, left panels). We tested for neutralization of HIV-1 IIIB and YU2 molecular clones. IIIB is a T-cell line adapted HIV-1 variant, which is highly sensitive to heterologous neutralization, while YU2 is a more resistant primary strain. No neutralization of YU2 was observed for any of the serum samples tested. Additionally, no neutralization of IIIB was detected for IIIB in serum samples up to day 1231 for MM1 and day 574 for MM4, while no neutralization of IIIB was detected at all for MM8 (last time point assayed was day 957). These observations indicate that variation in macrophage tropism occurred in the complete absence of nabs that target conserved epitopes including the CD4bs. To test for autologous nabs, we used the viral clones carrying gp120 sequences from early time points for MM1 (1.2.1, 1.2.3 from day 28 and 1.5.58 from day 84), MM4 (4.1.33 from day 17 and 4.4.48 from day 52) and MM8 (8.2.50, 8.2.51 from day 12 and 8.4.51 from day 32). We failed to detect autologous nabs against these viral clones until day 494 for MM1, day 206 for MM4 and day 81 for MM8 (Figure 2, right panels). Viral clones that varied in macrophage-tropism were therefore identified well before the detection of nabs in each of the three subjects.

HIV-1 R5 envelopes that vary dramatically in their capacities to infect macrophages were detected early after infection and long before nabs develop. For the three subjects investigated here, envelopes that varied in macrophage infection were detected at 28, 17 and 12 days respectively after the onset of acute stage symptoms. However, we were unable to detect autologous or heterologous nabs until many weeks or months later. The identification of macrophage-tropic variants early in disease seems at variance with the study by Salazar-Gonzalez *et al*. [25] who reported weak macrophage-tropism among 'so called' founder strains. However, such strains are believed to represent transmitted viruses rather than those from acute phase plasma studied here. Our study could be consistent with Isaacman-Beck's who showed a range of macrophage infectivity among the clade C envelopes, amplified from near acute phase plasma, although these authors did not investigate the envelopes' temporal relationship with nabs.

Our study shows that the selective pressures that confer variation in R5 mac-tropism in the early stages of infection do not involve nabs and thus remain unclear. However, there remains the possibility that ADCC and or complement mediated antibody functions play a role. In addition, when nabs do arise, they are likely to act as a selective pressure that impacts on tropism. This could be particularly true for heterologous nabs that target the CD4bs thus favouring non-mac-tropic variants that protect this site.

In summary, we show that variation in macrophage tropism observed at early times in HIV-1 infection has not been influenced by neutralizing antibodies.

## Competing interests

The authors declare that they have no competing interests.

## Authors' contributions

KHR carried out the macrophage and other infectivity assays and contributed to writing the manuscript. MMIAC and AM provided the molecular HIV-1 clones and MMIAC carried out the neutralization assays. PRC conceived the study and wrote the manuscript. MMIAC also helped interpreting the data and in editing the manuscript.
